# A Multicenter Study of Bacterial Vaginosis in Women With or at Risk for Human Immunodeficiency Virus Infection

**DOI:** 10.1155/S1064744901000242

**Published:** 2001

**Authors:** Dora Warren, Robert S. Klein, Jack Sobel, Burney Kieke, William Brown, Paula Schuman, Jean Anderson, Susan Cu-Uvin, Kenneth Mayer, Denise J. Jamieson, Scott Holmberg, Ann Duerr

**Affiliations:** 1 Centers for Disease Control and Prevention Atlanta GA USA; 2 Division of Infectious Diseases Department of Medicine and the Department of Epidemiology and Social Medicine Montefiore Medical Center and Albert Einstein College of Medicine Bronx NY USA; 3 Division of Infectious Diseases Wayne State University School of Medicine Department of Medicine Detroit MI USA; 4 Department of Pathology Wayne State University School of Medicine Detroit MI USA; 5 Division of Obstetrics and Gynecology Johns Hopkins School of Medicine Baltimore MD USA; 6 Division of Infectious Diseases Department of Medicine Miriam and Memorial Hospitals and Brown University School of Medicine Providence RI USA; 7 1600 Clifton Road, Mailstop E-45 Division of HIV/AIDS Prevention National Center for HIV, STD, TB Prevention Centers for Disease Control and Prevention Atlanta GA 30333 USA

## Abstract

*Background:* Bacterial vaginosis is a common gynecologic infection that has been associated with a variety of gynecologic and obstetric complications, including pelvic inflammatory disease, postabortal infection and premature delivery. Recent studies suggest that bacterial vaginosis may increase a woman’s risk for human immunodeficiency virus (HIV). We undertook this study to assess whether the prevalence and characteristics of bacterial vaginosis differed according to HIV status in high-risk US women.

*Methods:* Prevalence of bacterial vaginosis was assessed by Gram’s stain and clinical criteria for 854 HIV-infected and 434 HIV-uninfected women enrolled in the HIV Epidemiology Research (HER) Study.Multiple logistic regression techniques were used to determine whether HIV infection independently predicted bacterial vaginosis.

*Results: * Almost half (46%) the women had bacterial vaginosis by Gram’s stain. The prevalence of bacterial vaginosis was 47% in the HIV-positive women compared with 44% in the HIV-negativewomen; this difference was not statistically significant (p = 0.36). After adjustment for other covariates, HIV-positive women were more likely than HIV-negative women to have bacterial vaginosis (odds ratio (OR) 1.31; 95% confidence interval (CI) 1.01-1.70) by Gram's stain but not by clinical criteria (OR 1.16; CI 0.87-1.55). Among HIV-positive women, use of antiretroviral drugs was associated with a lower prevalence of bacterial vaginosis (adjusted OR 0.54; Cl 0.38 -0.77).

*Conclusions:* In this cross-sectional analysis of high-risk US women, HIV infection was positively correlated with bacterial vaginosis diagnosed by Gram’s stain.


### A multicenter study of bacterial vaginosis in women with or at risk for human immunodeficiency virus infection

**Dora Warren[1], Robert S. Klein[2], Jack Sobel[3], Burney Kieke, Jr[1], William Brown[4], Paula Schuman[3], Jean Anderson[5], Susan Cu-Uvin[6], Kenneth Mayer[6], Denise J. Jamieson[1], Scott Holmberg[1], Ann Duerr[1], for the HIV Epidemiology Research Study Group**

[1]*Centers for Disease Control and Prevention, Atlanta, GA*
[2]*Division of Infectious Diseases, Department of Medicine and the Department of Epidemiology and Social Medicine, Montefiore Medical Center and Albert Einstein College of Medicine, Bronx, NY*
[3]*Division of Infectious Diseases, Department of Medicine, Wayne State University School of Medicine, Detroit, MI*
[4]*Department of Pathology, Wayne State University School of Medicine, Detroit, MI*
[5]*Division of Obstetrics and Gynecology, Johns Hopkins School of Medicine, Baltimore, MD*
[6]*Division of Infectious Diseases, Department of Medicine, Miriam and Memorial Hospitals and Brown University School of Medicine, Providence, RI*

***Background:*** Bacterial vaginosis is a common gynecologic infection that has been associated with a variety of gynecologic and obstetric complications, including pelvic inflammatory disease, postabortal infection and premature delivery. Recent studies suggest that bacterial vaginosis may increase a woman's risk for human immunodeficiency virus (HIV). We undertook this study to assess whether the prevalence and characteristics of bacterial vaginosis differed according to HIV status in high-risk US women.
***Methods:*** Prevalence of bacterial vaginosis was assessed by Gram's stain and clinical criteria for 854 HIV-infected and 434 HIV-uninfected women enrolled in the HIV Epidemiology Research (HER) Study. Multiple logistic regression techniques were used to determine whether HIV infection independently predicted bacterial vaginosis.
***Results:*** Almost half (46%) the women had bacterial vaginosis by Gram's stain. The prevalence of bacterial vaginosis was 47% in the HIV-positive women compared with 44% in the HIV-negative women; this difference was not statistically significant ($p = 0.36$). After adjustment for other covariates, HIV-positive women were more likely than HIV-negative women to have bacterial vaginosis (odds ratio (OR) 1.31; 95% confidence interval (CI) 1.01–1.70) by Gram's stain but not by clinical criteria (OR 1.16; CI 0.87–1.55). Among HIV-positive women, use of antiretroviral drugs was associated with a lower prevalence of bacterial vaginosis (adjusted OR 0.54; CI 0.38 – 0.77).
***Conclusions:*** In this cross-sectional analysis of high-risk US women, HIV infection was positively correlated with bacterial vaginosis diagnosed by Gram's stain.

Key words: BACTERIAL VAGINOSIS; HIGH-RISK US WOMEN; HUMAN IMMUNODEFICIENCY VIRUS

---

Supported by cooperative agreements No. U64\CCU106795, U64\CCU206798, U64\CCU306802 and U64\CCU506831 with the Centers for Disease Control and Prevention
Correspondence to: Denise J. Jamieson, MD, MPH, 1600 Clifton Road, Mailstop E-45, Division of HIV/AIDS Prevention, National Center for HIV, STD, TB Prevention, Centers for Disease Control and Prevention, Atlanta, GA30333, USA. Email: djamieson@cdc.gov

Bacterial vaginosis, one of the most common gynecologic infections in women of reproductive age, has been associated with such important problems as premature delivery[1-4], endometritis and pelvic inflammatory disease[5-7]. In addition, bacterial vaginosis has been associated with increased risk of human immunodeficiency virus (HIV) acquisition among pregnant and postnatal women in Malawi[8].

Changes in the vaginal microbial ecosystem that characterize bacterial vaginosis involve overgrowth of *Gardnerella vaginalis*, *Bacteroides* spp., *Mobiluncus* spp. and genital mycoplasmas, as well as a reduction in *Lactobacillus* spp.[9,10]. Normal lactobacillus-dominant vaginal flora provide relative protection against genital pathogens by producing hydrogen peroxide ($H_2O_2$) and bacteriocins, and by maintaining a low vaginal pH[11,12]. The loss of these protective factors has been hypothesized to increase the risk of HIV acquisition in women with bacterial vaginosis. A cross-sectional study in Thailand of commercial sex workers attending a sexually transmitted disease (STD) clinic found that HIV infection was correlated with abnormal vaginal flora as well as clinical bacterial vaginosis[13]. Similarly, a study in Uganda found an association between HIV infection and bacterial vaginosis diagnosed by Gram's stain among women aged < 40 years[14]. More recently, a prospective study among pregnant and postnatal women in Malawi reported an increased risk of HIV acquisition among women with bacterial vaginosis[8].

To determine whether prevalence of bacterial vaginosis differs by HIV status in a high-risk US population, we studied HIV-infected and uninfected women participating in the HIV Epidemiology Research (HER) Study, which included sites in the Bronx, NY, Baltimore, MD, Detroit, MI and Providence, RI.

### METHODS

### Study population

Between April 1993 and January 1995 we recruited 1310 women to participate in the HER Study; eligibility requirements included being aged 16–55 years and having either a history of injection drug use since 1985 or sexual risk behaviors (e.g. > 5 sexual partners, trading sex for drugs or money, or sex with a male injection drug user or a man known or suspected to have HIV). HIV-positive women were only accepted if they did not have an autoimmune deficiency syndrome (AIDS)-defining clinical diagnosis[15]. As described elsewhere[16], women were recruited from health-care settings, drug treatment programs and social service organizations, and by word of mouth. The study was approved by the institutional review boards of each of the participating medical centers and the Centers for Disease Control and Prevention (CDC).

After informed consent was obtained, participants were administered a standardized face-to-face interview to obtain medical, obstetric and gynecologic histories, details of past and current drug use and sexual behaviors, and information on selected demographic and psychosocial factors. Following the interview, all women received a standardized physical examination and gave blood for HIV testing (enzyme-linked immunosorbent assay (ELISA) with confirmatory Western blot), immunophenotyping, syphilis serology and other laboratory tests.

### Gynecologic examination

All women underwent a standardized pelvic examination for diagnosis of gynecologic infection, which included an endocervical culture for *Neisseria gonorrhoeae*, a culture and direct fluorescent antibody test for *Chlamydia trachomatis*, a vaginal and anal culture for candida, and wet mounts (saline and potassium hydroxide) for *Trichomonas vaginalis*, clue cells and yeast. Vaginal pH was measured using indicator strips (Baxter S/P, Glendale, CA), and a whiff-amine test was performed by adding a drop of 10% potassium hydroxide to vaginal fluids and noting the release of a fishy odor. Signs and symptoms of vaginal infection at the time of physical examination were noted. A swab of the posterior vaginal fornix was used to prepare a smear that was air dried and fixed in methanol, then shipped to a central laboratory (Detroit Medical Center University Laboratories, Detroit, MI) for Gram's staining. Gram's stain slides were read by a single technologist who was unaware of the clinical or HIV status of participants. Under oil immersion (× 1000 magnification), slides were examined for the lactobacillus morphotypes, *Gardnerella* and *Bacteroides* spp. morphotypes (small Gram-negative to -variable rods) and *Mobiluncus* spp. morphotypes. Each of the three morphotypes was quantitated and scored as described by Nugent and colleagues[17]. For the lactobacillus morphotypes and *Gardnerella* and *Bacteroides* spp. morphotypes, the scores ranged from 0 to 4; for the *Mobiluncus* spp. morphotypes the score ranged from 0 to 2. The *Gardnerella*, *Bacteroides* and *Mobiluncus* spp. morphotypes were scored by giving those with the highest number of organisms the highest score. For the lactobacillus morphotypes the scoring was inversely related to the number of organisms present; those with the highest numbers of organisms received the lowest score. The scores from each of the three morphotypes were added together to get a range of possible scores from 0 to 10. A random sample ($n = 10$) of Gram's stain slides were read by an independent investigator (Carol Spiegel, University of Wisconsin, Madison, WI) with 100% agreement between the two readers.

### Definitions

The Gram's stain definition of bacterial vaginosis was used as the primary outcome for this analysis. The Gram's stain diagnosis was made according to the morphotype scoring system described above and developed by Nugent and colleagues[17]: a score of 0–3 was considered normal, 4–6 was considered intermediate and 7–10 was defined as bacterial vaginosis. In addition, a clinical diagnosis was defined using modified Amsel criteria[18]. The clinical diagnosis of bacterial vaginosis required that three of four conditions be present: vaginal pH > 4.7, abnormal vaginal discharge, positive whiff-amine test or any clue cells on microscopy.

Women with clinically diagnosed bacterial vaginosis were offered standard antibiotic therapy[19] at the time of examination.

### Statistical analysis

Analyses were conducted on data for the 1288 women (98.3% of the study group) who underwent a complete gynecologic examination. To investigate the correlates of bacterial vaginosis, separate analyses were conducted comparing women by Gram's stain result (score of 7–10 versus 0–6) and clinical diagnosis (yes, no). The $\chi^2$ or Fisher's exact test was used to compare proportions, and Student's *t*-test to compare means between groups. The Wilcoxon rank-sum test was employed for variables that were not normally distributed. Multiple logistic regression was used to identify correlates of bacterial vaginosis. Variables related to the study design were forced into all models, since their distributions were manipulated in the enrollment process. These included type of risk behavior (sexual or injected drug use), HIV status and the HER Study enrollment sites. Variables screened for possible inclusion in the multivariate models included covariates shown in previous studies to be associated with bacterial vaginosis or with HIV serostatus. In screening variables for inclusion in an initial multivariate model, models were fitted containing the study design variables and the variable being evaluated. Variables that exhibited at least moderate association ($p < 0.20$) with the outcome in the presence of these design variables were retained. Hence, factors included in the initial models were those related to the study design and those found to be associated with bacterial vaginosis in the screening process. First, multivariate models containing only main effects were fitted. Two-way interaction terms comprising HIV status and each variable retained in the main-effects-only model were subsequently examined. Variables retained in the final multivariate models included the study design variables, significant interaction terms (and the main effects that constituted them), main effects that attained statistical significance and variables whose exclusion meaningfully altered the adjusted odds ratio of any other variables retained in the model. In arriving at a final model, likelihood ratio tests were used to assess the significance of model terms and regression diagnostics were examined[20–23]. Because the high prevalence of bacterial vaginosis in the study population raised concerns that the odds ratio may not accurately approximate the relative risk, we calculated standardized prevalence ratios and associated 95% confidence intervals for the final Gram's stain bacterial vaginosis main-effects-only model using methods proposed by Flanders and Rhodes[24]. These methods were modified to accommodate multilevel exposures based on a conversation with Dr Rhodes. The marginal prediction method was used with the HER Study cohort serving as the standard population. All analyses were carried out using Statistical Analysis System (SAS) software[25].

### RESULTS

Of the 1288 women in our analysis, 854 were HIV-positive and 434 were HIV-negative. The median age of the women was 35 years; 58% were black, 24% were white and 16% were Hispanic. Over two-thirds (72%) had monthly household incomes less than \$1000, 43% had less than a high-school education and only 19% were employed at enrollment. Most (76%) were cigarette smokers and few were currently using hormonal contraceptives (5%) or spermicidal vaginal products (4%). None of these characteristics differed substantially according to HIV status. By design, about half (51%) of the women had a history of injection drug use since 1985, and the remainder (49%) reported only sexual risk factors for HIV; these proportions were similar in the HIV-positive and HIV-negative groups.

Overall, 46% (592/1281) of women showed bacterial vaginosis by Gram's stain and 35% (441/1268) met clinical criteria for bacterial vaginosis. In the unadjusted analyses, the prevalence of bacterial vaginosis by Gram's stain or by clinical diagnosis did not differ significantly according to HIV status (Table 1). In addition, there were no significant differences by Gram's stain in either the presence of abnormal vaginal flora (score 4–6) or the type of organisms (lactobacillus morphotypes, *Gardnerella* and *Bacteroides* spp. morphotypes, *Mobiluncus* spp. morphotypes) present.

Comparison by HIV status of the 592 women diagnosed with bacterial vaginosis by Gram's stain revealed similarities on numerous measures, including self-reported abnormal vaginal discharge and presence of *T. vaginalis* (Table 2).

In the multivariate analysis (Table 3), comparing women with a diagnosis of bacterial vaginosis (score 7–10) by Gram's stain with those having intermediate or normal vaginal flora (scores 0–6), HIV status was significantly associated with a positive diagnosis of bacterial vaginosis (adjusted odds ratio (OR) 1.31; 95% confidence interval (CI) 1.01–1.70). When an adjusted odds ratio was calculated by comparing women with a diagnosis of bacterial vaginosis (score 7–10) with those with normal vaginal flora (score 0–3), the magnitude of the association of HIV status with bacterial vaginosis was slightly greater; however, the confidence interval included 1.0 (adjusted OR 1.35; 95% CI 1.00–1.82: full model not shown).

Other covariates shown in the main multivariate model (Table 3) that were positively associated with prevalent bacterial vaginosis by Gram's stain include sex with a female within the last 6 months, number of male sexual partners in the last 6 months, sperm present on Gram's

**Table 1** Prevalence of bacterial vaginosis and abnormal vaginal flora by human immunodeficiency virus (HIV) status

| | *HIV+* (*n* = 854) | | *HIV–* (*n* = 434) | | |
|---|---|---|---|---|---|
| | n | % | n | % | p *value* |
| Gram's stain (score)* | | | | | |
| Bacterial vaginosis (7–10)† | 402 | 47 | 190 | 44 | 0.36 |
| Intermediate vaginal flora (4–6)† | 157 | 18 | 77 | 18 | 0.89 |
| Normal vaginal flora (0–3)† | 293 | 34 | 162 | 38 | 0.26 |
| Bacterial vaginosis, clinical‡ | 293 | 34 | 148 | 34 | 0.93 |
| Type of organisms by Gram's stain (> 6 per field) | | | | | |
| Lactobacillus morphotypes | 325 | 38 | 173 | 40 | 0.49 |
| *Gardnerella* and *Bacteroides* spp. morphotypes | 533 | 63 | 250 | 58 | 0.15 |
| *Mobiluncus* spp. morphotypes | 87 | 10 | 39 | 9 | 0.59 |

*For bacterial vaginosis by Gram's stain, there are seven missing values : five HIV– and two HIV+; †scores on Gram's stain according to system of Nugent and colleagues[17]; ‡for clinically diagnosed bacterial vaginosis, there are 20 missing values: five HIV– and 15 HIV+

**Table 2** Description of clinical characteristics of women diagnosed with bacterial vaginosis using Gram's stain criteria reported separately by human immunodeficiency virus (HIV) status

| | *HIV+* (*n* = 402) | | *HIV–* (*n* = 190) | | |
|---|---|---|---|---|---|
| | n | % | n | % | p *value** |
| Bacterial vaginosis, clinical† | 236 | 60‡ | 120 | 64‡ | 0.35 |
| Vaginal pH (median) | 5.3 | | 5.3 | | 0.37 |
| Abnormal vaginal discharge | | | | | |
| Self-report | 106 | 26‡ | 41 | 22 | 0.24 |
| On physical exam | 185 | 46 | 82 | 43‡ | 0.61 |
| Clue cells | | | | | |
| Wet mount | 236 | 61‡ | 117 | 64‡ | 0.64 |
| Gram's stain | 345 | 86 | 168 | 88 | 0.46 |
| *Trichomonas vaginalis* | 78 | 20‡ | 32 | 17‡ | 0.47 |

*$\chi^2$ Test for all *p* values except for vaginal pH where Wilcoxon rank-sum test is used; †by Amsel's criteria; ‡sample size decreased due to missing data

**Table 3** Unadjusted and adjusted risks of bacterial vaginosis diagnosed by Gram's stain criteria or by clinical criteria

| | *Bacterial vaginosis by Gram's stain** | | *Clinical bacterial vaginosis* | |
|---|---|---|---|---|
| | *Unadjusted OR† (95% CI)* | *Adjusted OR (95% CI)* | *Unadjusted OR† (95% CI)* | *Adjusted OR (95% CI)* |
| HIV-seropositive | 1.12 (0.89–1.42) | 1.31 (1.01–1.70) | 1.01 (0.78–1.31) | 1.16 (0.87–1.55) |
| Sex with female in last 6 months | 1.74 (1.14–2.67) | 1.82 (1.14–2.90) | 1.51 (0.96–2.36) | 1.70 (1.04–2.78) |
| Number of male sex partners in last 6 months | | | | |
| None | Referent | Referent | Referent | Referent |
| 1 | 1.36 (1.02–1.81) | 1.39 (1.02–1.91) | 1.36 (0.99–1.88) | 1.41 (0.99–2.00) |
| 2 or more | 1.79 (1.27–2.51) | 1.73 (1.21–2.48) | 2.12 (1.46–3.08) | 2.07 (1.40–3.08) |
| Black race | 1.64 (1.24–2.17) | 1.57 (1.17–2.12) | 2.24 (1.64–3.06) | 2.11 (1.51–2.93) |
| Sperm present on Gram's stain | 2.31 (1.57–3.41) | 2.28 (1.51–3.44) | — | — |
| Smoked cigarettes in last 6 months | 1.43 (1.08–1.88) | 1.35 (1.01–1.82) | 2.02 (1.47–2.79) | 1.67 (1.20–2.35) |
| Vaginal candida colonization | 0.55 (0.43–0.71) | 0.50 (0.39–0.66) | 0.76 (0.58–0.99) | 0.72 (0.54–0.96) |
| *Trichomonas vaginalis* | 2.28 (1.58–3.31) | 2.17 (1.47–3.19) | 3.59 (2.45–5.25) | 3.36 (2.26–4.99) |
| Vulvar, vaginal or cervical condyloma | 1.58 (1.10–2.26) | 1.61 (1.10–2.34) | — | — |
| Menses within last 14 days | — | — | 1.36 (1.06–1.75) | 1.30 (0.99–1.70) |
| Ectopy present | — | — | 1.48 (0.99–2.21) | 1.58 (1.04–2.40) |

*Bacterial vaginosis by Gram's stain defined as Gram score of 7–10 compared with referent group, 0–6; †adjusted only by study design variables: site, risk cohort and human immunodeficiency virus (HIV) status; OR, odds ratio; CI, confidence interval stain, smoking cigarettes in the past 6 months, *T. vaginalis* by culture or wet mount, condyloma on the vulva, vagina or cervix, and black race. Vaginal candida colonization by culture was negatively associated with prevalent bacterial vaginosis by Gram's stain (adjusted OR 0.50; 95% CI 0.39–0.66).

A multivariate analysis comparing women with a clinical diagnosis of bacterial vaginosis with those without a clinical diagnosis was also performed (Table 3). The unadjusted and adjusted odds ratios were all in a similar direction to those in the multivariate analysis using Gram's stain for diagnosis. However, the associations with some covariates did not reach statistical significance, most notably the association of HIV seropositivity (adjusted OR 1.16; 95% CI 0.87–1.55).

The association of the racial covariate was explored in more detail. A model was constructed that included as covariates additional measurements of socioeconomic status collected for this study, including birth outside the United States, not residing in own home or apartment, no current employment, currently receiving public assistance and total monthly household income of < $1000. In this model the relationship of bacterial vaginosis by Gram's stain and black race was not substantially altered (adjusted OR 1.70; 95% CI 1.23–2.35: full model not shown).

For the main multivariate model, significant interaction was found between HIV serostatus and vaginal candida colonization. Among women with a positive candida culture, there was a significant association of bacterial vaginosis by Gram's stain with HIV seropositivity (adjusted OR 2.85; 95% CI 1.58–5.13). Among women with a negative candida culture, there was no significant association with HIV seropositivity (adjusted OR 1.05; 95% CI 0.78–1.42), although the association was in the same direction.

In a multivariate model restricted to HIV-positive women (Table 4), antiretroviral drug use was protective against bacterial vaginosis by Gram's stain (adjusted OR 0.54; 95% CI 0.38–0.77). There was no significant association of CD4 group with bacterial vaginosis in this model.

### DISCUSSION

In this cross-sectional analysis of women with or at risk for HIV infection, we found an increased risk of bacterial vaginosis diagnosed by Gram's stain among women with HIV infection (adjusted OR 1.31; 95% CI 1.01–1.70). These findings, consistent with two previous reports from Thailand and Uganda[13,14], are among the first reported for US women being studied at other than clinical visits. Among women with HIV, a lower CD4 count was associated with an increased risk of bacterial vaginosis, although this association was not significant. Current antiretroviral use, however, was associated with a decreased prevalence of bacterial vaginosis.

The prevalence of bacterial vaginosis in this group of women with or at risk for HIV infection was very high, particularly as specimens were obtained during pre-scheduled study visits that were not symptom driven. Almost half (46%) the women had bacterial vaginosis by Gram's stain. These high rates of bacterial vaginosis are similar to findings in three studies of women attending sexually transmitted disease clinics (range 12–61%)[26–28], but are generally higher than reports

**Table 4** Unadjusted and adjusted risks of bacterial vaginosis diagnosed by Gram's stain criteria among human immunodeficiency virus (HIV)-positive women

| | *Bacterial vaginosis by Gram's stain** | |
|---|---|---|
| | *Unadjusted OR† (95% CI)* | *Adjusted OR (95% CI)* |
| CD4 group | | |
| > 500 | Referent | Referent |
| 200–500 | 1.06 (0.78–1.45) | 1.33 (0.93–1.91) |
| < 200 | 0.95 (0.63–1.44) | 1.47 (0.92–2.35) |
| Current antiretroviral use | 0.56 (0.41–0.75) | 0.54 (0.38–0.77) |
| Sex with female in last 6 months | 2.46 (1.36–4.46) | 2.42 (1.26–4.63) |
| Number of male sex partners in last 6 months | | |
| None | Referent | Referent |
| 1 | 1.39 (1.00–1.94) | 1.41 (0.98–2.04) |
| 2 or more | 2.08 (1.36–3.18) | 1.98 (1.25–3.14) |
| Black race | 1.61 (1.14–2.29) | 1.40 (0.96–2.04) |
| Sperm present on Gram's stain | 2.53 (1.54–4.14) | 2.43 (1.43–4.14) |
| Smoked cigarettes in last 6 months | 1.41 (1.01–1.96) | 1.46 (1.01–2.10) |
| Vaginal candida colonization | 0.69 (0.52–0.92) | 0.59 (0.43–0.81) |
| *Trichomonas vaginalis* | 2.42 (1.54–3.81) | 2.18 (1.35–3.52) |
| Vulvar, vaginal or cervical condyloma | 1.52 (1.02–2.26) | 1.49 (0.97–2.28) |

*Bacterial vaginosis by Gram's stain defined as Gram score of 7–10 compared with referent group, 0–6; †adjusted only by study design variables: site and risk cohort; OR, odds ratio; CI, confidence interval for pregnant women (range 10–32%)[3,29,30] and above those for college students (range 4–23%)[28,31]. As has been reported in other studies[3,9,28], most bacterial vaginosis was asymptomatic in this population, with less than one-quarter (24.8%) of women reporting abnormal vaginal discharge.

We chose to define bacterial vaginosis primarily by Gram's stain using the criteria of Nugent and colleagues[17], since it is generally thought to be more reliable than wet mount[32] and has been used in a number of recent studies[33,34]. A smaller percentage of women (34%) met the clinical criteria for bacterial vaginosis, compared with the Gram's stain diagnosis. The positive predictive value of clinical diagnosis compared with Gram's stain was similar for HIV-positive and HIV-negative women: 80.8% and 81.6%, respectively.

As in previous reports[35,36], we found that candida was protective against bacterial vaginosis. Because there was significant interaction between HIV serostatus and vaginal candida colonization, we explored this relationship further. We found that the association of HIV seropositivity with bacterial vaginosis was significant among women with a positive candida culture and not significant (although in the same direction) among women with a negative candida culture. Since more HIV-infected women have candida, and since a positive candida culture is somewhat protective against bacterial vaginosis, this explains in part why the unadjusted odds ratio for HIV-seropositivity was not significant and the adjusted odds ratio was significant. It has been suggested that the overgrowth of anaerobic bacteria and increased pH associated with bacterial vaginosis infection may inhibit the growth of candida[36].

In addition to candida, several other correlates of bacterial vaginosis were identified in this study. Consistent with several previous reports[3,4], we found that women who were positive for bacterial vaginosis were more likely to be of black race. Because race may be a marker for many underlying social, economic, cultural and behavioral factors[37], we attempted to control for as many of these factors as we were able to measure in this study. When we included in the multivariate model all of the socioeconomic factors we collected, the relationship between race and bacterial vaginosis was not substantially altered, suggesting that race may be a marker for an underlying factor that we were not able to measure. Alternatively, race may be a marker for the prevalence of bacterial vaginosis in a particular community.

In this study, characteristics significantly related to the presence of bacterial vaginosis included having sex with a female within the last 6 months, having more male sex partners in the last 6 months, having sperm present on the Gram's stain, smoking cigarettes within the last 6 months, having *T. vaginalis* and having genital condylomata. Most of these covariates are related to more frequent sexual activity, and a similar relationship with number of male partners was found in a study from Malawi. Additionally, the Malawi study also reported increased prevalence of bacterial vaginosis when *T. vaginalis* was present. The relationship with having had sex with a female is consistent with some evidence that female-to-female transmission of bacterial vaginosis occurs. In a small study of 21 lesbian couples, 72% of partners of women with bacterial vaginosis also had bacterial vaginosis, compared with only 10% of partners of those without bacterial vaginosis[38].

The limitations of this study should be taken into account when interpreting the results. One of the goals of the HER study was to assess the effects of HIV on gynecologic infection independent of other risk factors such as drug use or sexual behaviors; therefore, by design, all HIV-uninfected HER study participants were at increased risk for HIV and not necessarily representative of all women without HIV. Thus, generalizability of the results of this study to the general population may be limited. In addition, this analysis was cross-sectional, and therefore cannot evaluate the incidence or persistence of bacterial vaginosis.

In contrast to prior studies in which evaluations were performed at clinical visits, this study evaluated women at scheduled research visits, thereby minimizing the potential biases associated with symptom-driven evaluations. In addition, there was multiple ascertainment of the diagnosis of bacterial vaginosis (Gram's stain and clinical criteria) in this study, with consistency of results.

Given our finding that HIV-infected women are more likely than uninfected women to have prevalent bacterial vaginosis, it will be important to examine more closely this association longitudinally, to determine whether HIV-infected women have a higher incidence of infection or whether they have more persistent infections, or both. This association may have important implications in terms of treatment of bacterial vaginosis and prevention of HIV acquisition.

### ACKNOWLEDGEMENTS

We thank all of the women who participated in the HER Study, the study staff and Karen Manning for her assistance in the preparation of this manuscript.

### Members of the HER Study Group

The HER Study group consists of the authors and Ellie Schoenbaum, MD, Julia Arnsten, MD, MPH, Penelope Demas, PhD, and Andrea Howard, MD, MSc, from Montefiore Medical Center and the Albert Einstein College of Medicine; Suzanne Ohmit, DrPH, Michael Long PhD, Wayne Lancaster PhD, and Jose Vazquez, MD, from the Wayne State University School of Medicine; Anne Rompalo, MD, David Vlahov, PhD and David Celentano, PhD, from the Johns Hopkins University School of Medicine; Charles Carpenter, MD, Timothy Flanigan, MD, Joseph Hogan, ScD, Valerie Stone, MD, Karen Tashima, MD, and Josiah Rich, MD, from the Brown University School of Medicine; Lytt I. Gardner, PhD, Chad Heilig, PhD, Janet S. Moore, PhD, Ruby M. Phelps, BS, and Dawn K. Smith, MD, MPH, from the Centers for Disease Control and Prevention; and Katherine Davenny, MPH, from the National Institute of Drug Abuse.

### REFERENCES

1. Gibbs RS. Chorioamnionitis and bacterial vaginosis. *Am J Obstet Gynecol* 1993;169:460–2
2. Hauth JC, Goldenberg RL, Andrews WW, *et al.* Reduced incidence of preterm delivery with metronidazole and erythromycin in women with bacterial vaginosis. *N Engl J Med* 1995;333:1732–6
3. Hillier SL, Nugent RP, Eschenbach DA, *et al.* Association between bacterial vaginosis and preterm delivery of a low-birth-weight infant. The Vaginal Infections and Prematurity Study Group. *N Engl J Med* 1995;333:1737–42
4. Meis PJ, Goldenberg RL, Mercer B, *et al.* The preterm prediction study: significance of vaginal infections. National Institute of Child Health and Human Development Maternal–Fetal Medicine Units Network. *Am J Obstet Gynecol* 1995;173: 1231–5
5. Larsson PG, Platz-Christensen JJ, Thejls H, *et al.* Incidence of pelvic inflammatory disease after first-trimester legal abortion in women with bacterial vaginosis after treatment with metronidazole: a double-blind, randomized study. *Am J Obstet Gynecol* 1992;166:100–3
6. Padian NS, Washington AE. Pelvic inflammatory disease. A brief overview. *Ann Epidemiol* 1994;4: 128–32
7. Sweet RL. Role of bacterial vaginosis in pelvic inflammatory disease. *Clin Infect Dis* 1995;20 (Suppl 2):S271–5
8. Taha TE, Hoover DR, Dallabetta GA, *et al.* Bacterial vaginosis and disturbances of vaginal flora: association with increased acquisition of HIV. *AIDS* 1998;12:1699–706
9. Sobel JD. Vaginitis in adult women. *Obstet Gynecol Clin North Am* 1990;17:851–79
10. Hillier SL. Diagnostic microbiology of bacterial vaginosis. *Am J Obstet Gynecol* 1993;169:455–9
11. Hillier SL, Krohn MA, Rabe LK, *et al.* The normal vaginal flora, $H_2O_2$-producing lactobacilli, and bacterial vaginosis in pregnant women. *Clin Infect Dis* 1993;16 (Suppl 4):S273–81
12. Klebanoff SJ. Inactivation of human immunodeficiency virus type 1 by the amine oxidase–peroxidase system. *J Clin Microbiol* 1995;33:2054–7
13. Cohen CR, Duerr A, Pruithithada N, *et al.* Bacterial vaginosis and HIV seroprevalence among female commercial sex workers in Chiang Mai, Thailand. *AIDS* 1995;9:1093–7
14. Sewankambo N, Gray RH, Wawer MJ, *et al.* HIV-1 infection associated with abnormal vaginal flora morphology and bacterial vaginosis. *Lancet* 1997;350:546–50
15. Centers for Disease Control and Prevention. 1993 Revised classification system for HIV infection and expanded surveillance case definition for AIDS among adolescents and adults. *Morbid Mortal Weekly Rep* 1992;41(RR-17):1–19

16. Smith DK, Warren D, Vlahov D, *et al*. The design, participants, and selected early findings of the HIV Epidemiology Research (HER) Study: a prospective cohort study of the impact of HIV infection on the health of American women. *Am J Epidemiol* 1997;146:459–69
17. Nugent RP, Krohn A, Hillier SL. Reliability of diagnosing bacterial vaginosis is improved by a standardized method of Gram stain interpretation. *J Clin Microbiol* 1991;29:297–301
18. Amsel RM, Totten PA, Spiegal CA, *et al*. Nonspecific vaginitis: diagnostic criteria and microbial and epidemiologic associations. *Am J Med* 1983;74:14–22
19. Centers for Disease Control and Prevention. 1993 Sexually transmitted diseases treatment guidelines. *Morbid Mortal Weekly Rep* 1993;42(RR-14):68–70
20. Belsley DA, Kuh E, Welsch RE. *Regression Diagnostics*. New York: John Wiley & Sons, 1980
21. Davis CE, Hyde JE, Bangdiwala SI, Nelson JJ. An example of dependencies among variables in a conditional logistic regression. In Moolgavkar SH, Prentice RL, eds. *Modern Statistical Methods in Chronic Disease Epidemiology*. New York: John Wiley & Sons, 1986:140–7
22. Hosmer DW, Lemeshow S. *Applied Logistic Regression*. New York: John Wiley & Sons, 1989:140–5
23. Pregibon D. Logistic regression diagnostics. *Ann Stat* 1981;9:705–24
24. Flanders WD, Rhodes PH. Large sample confidence intervals for regression standardized risks, risk ratios, and risk differences. *J Chron Dis* 1987;40:697–704
25. SAS Institute Inc. *SAS/STAT Software: Changes and Enhancements through Release 6.12*. Cary, NC: SAS Institute Inc., 1997:1–1167
26. Centers for Disease Control. Nonreported sexually transmissible disease – United States. *Morbid Mortal Weekly Rep* 1979:28:61–3
27. Embree J, Caliando JJ, McCormack WM. Nonspecific vaginitis among women attending a sexually transmitted diseases clinic. *Sex Transm Dis* 1984;11:81–4
28. Eschenbach DA, Hillier S, Critchlow C, *et al*. Diagnosis and clinical manifestations of bacterial vaginosis. *Am J Obstet Gynecol* 1988;158:819–28
29. Gravett MG, Nelson HP, DeRouen T, *et al*. Independent associations of bacterial vaginosis and *Chlamydia trachomatis* infection with adverse pregnancy outcome. *J Am Med Assoc* 1986;225:1899–903
30. Minkoff H, Grunebaum AN, Schwarz RH, *et al*. Risk factors for prematurity and premature rupture of membranes: prospective study of vaginal flora in labor. *Am J Obstet Gynecol* 1984;150:965–72
31. Spiegel CA, Amsel R, Eschenbach D, *et al*. Anaerobic bacteria in nonspecific vaginitis. *N Engl J Med* 1980;303:601–7
32. Sobel JD. Vaginitis. *N Engl J Med* 1997;337:1896–903
33. Carey JC, Klebanoff MA, Hauth JC, *et al*. Metronidazole to prevent preterm delivery in pregnant women with asymptomatic bacterial vaginosis. *N Engl J Med* 2000;342:534–9
34. Royce RA, Thorp J, Granados JL, Savitz DA. Bacterial vaginosis associated with HIV infection in pregnant women from North Carolina. *J AIDS* 1999;20:382–6
35. Taha TE, Gray RH, Kumwenda NI, *et al*. HIV infection and disturbances of vaginal flora during pregnancy. *J AIDS* 1999:20:52–9
36. Martin HL, Richardson BA, Nyange PM, *et al*. Vaginal lactobacilli, microbial flora, and risk of human immunodeficiency virus type 1 and sexually transmitted disease acquistion. *J Infect Dis* 1999;180:1863–8
37. Centers for Disease Control. *Morbid Mortal Weekly Rep* 1993;42(RR-10):
38. Berger BJ, Kolton S, Zenilman JM, *et al*. Bacterial vaginosis in lesbians: a sexually transmitted disease. *Clin Infect Dis* 1995;21:1402–5

RECEIVED 4/23/01; ACCEPTED 5/28/01
